# Improving blood culture procurement: a prospective 5-year hospital-wide study

**DOI:** 10.1186/s13584-025-00744-x

**Published:** 2025-12-31

**Authors:** Shmuel Benenson, Miriam Dreyer, Bath Sheva Ezagui, Ilana Dery, Todd Zalut, Naama Bagrish, Rona Lujan, Marc V. Assous, Amos M. Yinnon, Oshrat Ayalon

**Affiliations:** 1 The Infection Control and Prevention Unit, Shaare Zedek Medical Center, P.O. Box 3235, Jerusalem, Israel; 2 The Emergency Department, Shaare Zedek Medical Center, Jerusalem, Israel; 3 The Eisenberg R&D Authority, Shaare Zedek Medical Center, Jerusalem, Israel; 4 The Clinical Microbiology Laboratory, Shaare Zedek Medical Center, Jerusalem, Israel; 5 The Medical Center is affiliated with the Hebrew-University Hadassah Medical School, Jerusalem, Israel

**Keywords:** Blood cultures, Contaminants, Outcome measures, Guideline adherence, Bundle approach

## Abstract

**Background:**

Appropriate procurement of blood cultures (BC) is essential for diagnosis of bacteremia and susceptibility testing. This includes (1) adequate preparation of the venipuncture site to minimize contamination; (2) obtaining ≥ two sets with a time interval before starting antibiotics. Although these recommendations are standard since the 1960s, adherence is far less than expected – which may adversely impact on the management of bacteremic patients.

**Aims of study:**

This single-center study conducted in Shaare Zedek Medical Center aimed to decrease the proportion of contaminated BCs and to increase the percentage of obtaining two sets of BC/ blood-culture-taking episode.

**Methods:**

Determination of both markers at baseline, then monthly for one year, then subsequently on a quarterly basis; showing data from all departments in real-time to all department directors; and providing short educative lectures during departmental staff meetings, at baseline and after 1–2 years. These markers were adopted as one of the hospital-wide quality measures.

**Results:**

In the 20-year period 2000–2019 more than 1 million BCs were obtained, of which 70% were from patients ≤ 72 h in hospital. During the 5-year study (2020–2024), the percent of blood-culture-taking episodes from which two culture sets were obtained increased annually by ± 16% from a baseline of 27% (9010/33306) in 2020, to 46% (18462/40191) in 2024 (Incidence Rate Ratios, IRR 1.16 [95%CI 1.13–1.18], *p* < 0.001). This improvement was observed in almost all departments and was especially profound in the emergency department (ED), starting at a baseline of 19% (1979/10326) and increasing to 53% (5304/9915)(IRR 1.33 [95%CI 1.27–1.39], *p* < 0.001). During the same period, the annual proportion of false-positive BCs, from which only contaminants were isolated, decreased annually by 18% from 2.4% (1592/65230) in 2020 to 1.3% (895/68991) in 2024 (IRR 0.82 [95%CI 0.77–0.88], *p* < 0.001). This improvement was observed in all departments: in the emergency department, this rate decreased from 3.3% (676/20529) to 1.56% (272/17459) (IRR 0.79 [95%CI 0.75–0.83], *p* < 0.001).

**Conclusion:**

A simple educational intervention, combined with meticulous data mining and presentation of each department’s results, with comparison of all other departments, led to significant and sustained improvement in measurable markers.

## Introduction

Although the main components of appropriately obtaining blood specimens for culture in patients with suspected sepsis have been developed decades ago [1–3], there continues to be a significant gap between guidelines and real-life practice [4–7]. The three features that impact on the outcomes most significantly are adequate skin preparation to prevent contamination, obtaining at least two sets prior to antibiotic initiation, and sufficient volumes of blood. Although these principles are taught during the basic training of all medical students and nurses, multiple studies have demonstrated poor adherence to guidelines [7–9], with disappointingly high rates of contamination and lower than expected rates of true positive cultures.

The digitalization of microbiology laboratory culture results allows for big data analysis of appropriate collection of blood culture samples. A 20-year (2000–2019) retrospective analysis of 1,149,603 blood cultures in our medical center showed a true positive and contamination rate of, respectively 7.3% and 3.7%. In addition, a second blood culture was obtained from only 27% of patients from whom a first blood culture was obtained in the emergency department, and from only 44% of patients from whom a first culture was obtained in the hospital’s departments. These disappointing baseline data prompted the current study, with the twofold objective of reducing contamination rates and increasing the percentage of septic patients from whom two sets of blood cultures were obtained.

## Methods

### Setting

This study was performed in Shaare Zedek Medical Center, a 1000-bed general, university-affiliated medical center in Jerusalem, Israel. The hospital provides all medical services, except solid organ transplantations, but allogeneic bone marrow transplantations are performed. The medical division includes four medical departments and one acute-care geriatric department for a total of ± 250 beds, a 25-bed oncology department and a 25-bed hematology department. The cardiac division includes a 45-bed in-patient department. In addition, there are the usual medical subspecialty units (including nephrology/dialysis, rheumatology, infectious diseases, pulmonology, endocrinology, Gaucher, etc.). There are six Intensive Care Units (ICUs): a 14-bed medical-surgical ICU, Intensive Cardiac Care (10 beds), Pediatric Intensive Care (6 beds), Neurosurgical Intensive Care (4 beds), Cardiac Surgery (4 beds) and Neonatal Intensive Care (15 beds). The general surgical department includes > 70 beds, and the obstetrics department handles annually > 22.000 deliveries. In addition, there are the usual surgical subspecialty departments (orthopedics, plastic surgery, ophthalmology, vascular surgery, urology, ear-nose-throat, etc.). The pediatric hospital contains a 35-bed in-patient department and all pediatric sub-specialties. There are three busy emergency departments: general emergency, pediatric emergency and obstetrics/gynecology emergency.

The infection control and prevention unit: consists of two physicians, of whom one serves as director and seven infection control practitioners (ICP), all of whom have completed a one-year infection control program and have passed national board examinations. All ICPs are assigned to several clinical departments where they round several times each week.

### Blood culture guidelines

As in most hospitals, physicians in all departments may obtain blood cultures at their own discretion. Standard recommendations include (1) adequate disinfection of the venipuncture site with an alcohol impregnated swab; (2) obtaining two sets prior to initiation of antibiotic treatment, preferably > half an hour apart and, if possible, from two different venipuncture sites; (3) each set should contain an aerobic and anaerobic bottle, each of which should be filled with 7–10 ml; (4) the obtained culture bottles should be sent immediately to the microbiology laboratory by the pneumatic system. Unfortunately, in our medical center like most Israeli hospitals, the microbiology laboratory is not yet staffed during nightshifts.

### Time frame

The project reported in this paper started in 2020 and continues. In this article we describe the findings of the first five years of the project (2020-24) and provide the background data from the period 2000–2019.

### Definition

For this study we defined a “blood-culture-taking episode” as one when any clinician decided, for whatever indication, to obtain a blood culture set. We expected that another set was obtained < 24 h after the first set. Ideally, the second set should be obtained after a reasonable time interval, between half an hour and two hours, depending on the patient’s clinical condition, prior to initiation of antibiotic treatment, although the computer system permitted a definition of < 24 h, two consecutive calendar dates.

### Aim of the current study

To improve the quality of blood culture procurement by educational intervention. The quality of blood culture procurement can be measured by three objective markers: first, the percentage of blood-culture-taking episodes from which two culture sets (i.e., 4 bottles) were obtained at different time intervals within 24 h, preferably before initiation (or change) of antibiotics; second, the percentage of false positive cultures, from which contaminant organisms were isolated (see below definitions); third, the volume of collected blood in the culture bottles. We selected the first two markers for follow-up, as these are computerized and accessible, whereas the measurement of volume of collected blood in the culture bottles is not yet automated.

### Interventions

The infection Control and Prevention Unit, together with the Clinical Microbiology Laboratory, selected the quality of blood culture procurement as an important, hospital-wide, clinical outcome marker in 2021, for several reasons. First, the baseline percentage of collection of two blood cultures before initiation of antibiotic treatment was determined and found to be consistently low (< 30%), probably leading to underdiagnosis of bacteremias with possibly suboptimal consequences for the relevant patients. Second, the rate of blood cultures with contaminants was found to be unacceptably high (> 2%), leading to a waste of resources in the Microbiology Laboratory and, quite likely, significant unnecessary prescription of antimicrobial treatment.

The educational intervention consisted of a baseline 10–15-minute presentation in all hospital departments. The presentation included, first, the standard guideline recommendations for obtaining blood cultures; second, baseline data of the entire hospital as well as those of the relevant department; and third, practical advice on how to improve clinical practice. All staff members were made aware of the fact that follow-up data would be collected, initially monthly and then quarterly, and would be shown to the relevant teams as well as to the entire hospital’s staff. One and a half years later, a similar follow-up lecture was given to all departments which obtained > 25 sets of blood cultures per quarter. The infection control practitioners were instructed to include occasional reference to appropriate blood culture procurement while conducting their educational rounds in the hospital’s departments.

### Microbiology

Blood cultures were incubated using BACTEC™ FX systems (BD, Franklin lakes, NJ, USA). Positive blood cultures were sub-cultured onto chocolate agar, blood agar, MacConkey agar, phenylethyl alcohol (PEA) agar, and CNA agar for anaerobes, using Becton Dickinson (BD, USA) and HyLabs (Rehovot, Israel) media. Identification of bacterial colonies was performed using matrix-assisted laser desorption/ionization time-of-flight mass spectrometry (MALDI-TOF MS) with the Bruker Biotyper system (Bruker Daltonics, Bremen, Germany). We designated the following organisms as obvious external contaminants unless isolated from two different blood culture sets: coagulase-negative *Staphylococcus*, *Micrococcus*, alpha hemolytic *Streptococcus*, Coryneform Gram positive rods and *Bacillus* [10].

### Ethics

This study was conducted as a hospital-wide quality improvement project. No patient-related interventions were performed and no patient data were collected. Accordingly, no formal ethics approval was deemed mandatory.

### Duration of the study, data extraction and analysis

All data were retrieved through the Medical Center’s computer system. Since 1997, all microbiology data are entered into computer applications, which by April 2025 contained data of 2.5 million cultures. For background we included data from all blood cultures, obtained over two decades (2000–2019). We performed an interrupted time series analysis, using Poisson regression to model quarterly and yearly incidence rates with an offset for the number of blood culture taking episodes. The dependent variables were (1) the rate of obtaining two blood culture sets per episode and (2) the contamination rate. The model included terms for time, indication for period and an interaction term between time and intervention. We performed two analyses, the first based on quartiles, the second based on whole years. Although the trend analysis for the two analyses was similar, the larger numbers of obtained blood cultures shown for each point in time in the figure with annual (as compared to the quartile) data led us to select the former for inclusion in the paper, as shown in the two figures. As the main intervention was made in 2020 (with data from 2000 to 2019) serving as baseline, five post-intervention points are shown. Incidence rate ratios (IRRs) with 95% confidence intervals were calculated for data accrued between 2020 and 2024 regarding rate of obtainment of two blood culture sets per blood-culture-taking episode and contamination rate. An IRR greater than 1 indicates an increase in rates over time, while an IRR less than 1 indicates a decrease. Model assumptions were checked, and a robust variance estimator was applied.

## Results

In the 20-year period 2000–2019 more than one million blood cultures were obtained (*n* = 1,149,603), of which 70% were from patients ≤ 72 h in hospital (usually indicating suspected community acquired infections) (Table 1). True positive organisms were isolated from 6.1% of patients ≤ 72 h in hospital as compared to 10.1% of patients > 72 h in hospital (Diff = −4%, 95%CI [−4.1, −3.9%], *p* < 0.001). Contaminants were isolated from 2.8% of cultures from patients ≤ 72 h in hospital, as compared to 5.4% contaminants from cultures obtained > 72 h in hospital (Diff = −2.6%, 95%CI [−2.7%, −2.5%], *p* < 0.001). A second blood culture was obtained from only 27% of patients from whom a first set was obtained in the emergency department, and from 44% of patients in the hospital’s departments (Diff = −17%, 95%CI [−17.2%, −2.5%), *p* < 0.001).

**Table 1:** Number of obtained blood cultures (n), rate of obtaining two sets and rates of true and false positivity (%), community acquired versus hospital acquired (2000–2019)

Departments/Obtained Blood Cultures (*n*)	Two Blood Cultures obtained %	True Positive *n* (%)	False Positive *N* (%)
Emergency Department (352,822)	112,903 (32)	26,814 (7.6)	11,643 (3.3)
≤72 h in hospital (339,445)	91,659 (27)	25,119 (7.4)	11,202 (3.3)
>72 h (11,199)	4,928 (44)	1,120 (10.0)	414 (3.7)
Intensive Care Unit (42,558)	24,684 (58)	5,532 (13.0)	3,617 (8.5)
≤72 h in hospital (13,048)	8,481 (65)	1,240 (9.5)	848 (6.5)
>72 h (29,463)	15,910 (54)	4,272 (14.5)	2,769 (9.4)
Medical Departments (253,966)	1,142,845 (45)	28,190 (11.1)	12,444 (4.9)
≤72 h in hospital (74,949)	38,973 (52)	(9,518) 12.7	2998 (4.0)
>72 h (171,552)	72,052 (42)	17,498 (10.2)	9,092 (5.3)
Pediatrics (359,263)	50,297 (14)	12,933 (3.6)	10,419 (2.9)
≤72 h in hospital (266,554)	23,990 (9)	6,664 (2.5)	4,798 (1.8)
>72 h (27,194)	12,509 (46)	1,985 (7.3)	1,115 (4.1)
Surgery (112,393)	41,585 (37)	89,91 (8.0)	4,046 (3.6)
≤72 h in hospital (42,714)	13,241 (31)	2,648 (6.2)	812 (1.9)
>72 h (69,428)	29,160 (42)	6,248 (9.0)	3,194 (4.6)
All Hospital (1,149,603)	367,873 (32)	83,921 (7.3)	42,535 (3.7)
≤72 h in hospital (749,228)*	202,292 (27)	45,793 (6.1)	20,978 (2.8)
>72 h (312,901)*	137,676 (44)	31,603 (10.1)	16,897 (5.4)

In addition to blood cultures from admitted patients, 87,474 blood cultures were obtained

in the out-patient clinics and from staff members. Together, these add up to 1,149,603 blood cultures

**Fig. 1:**
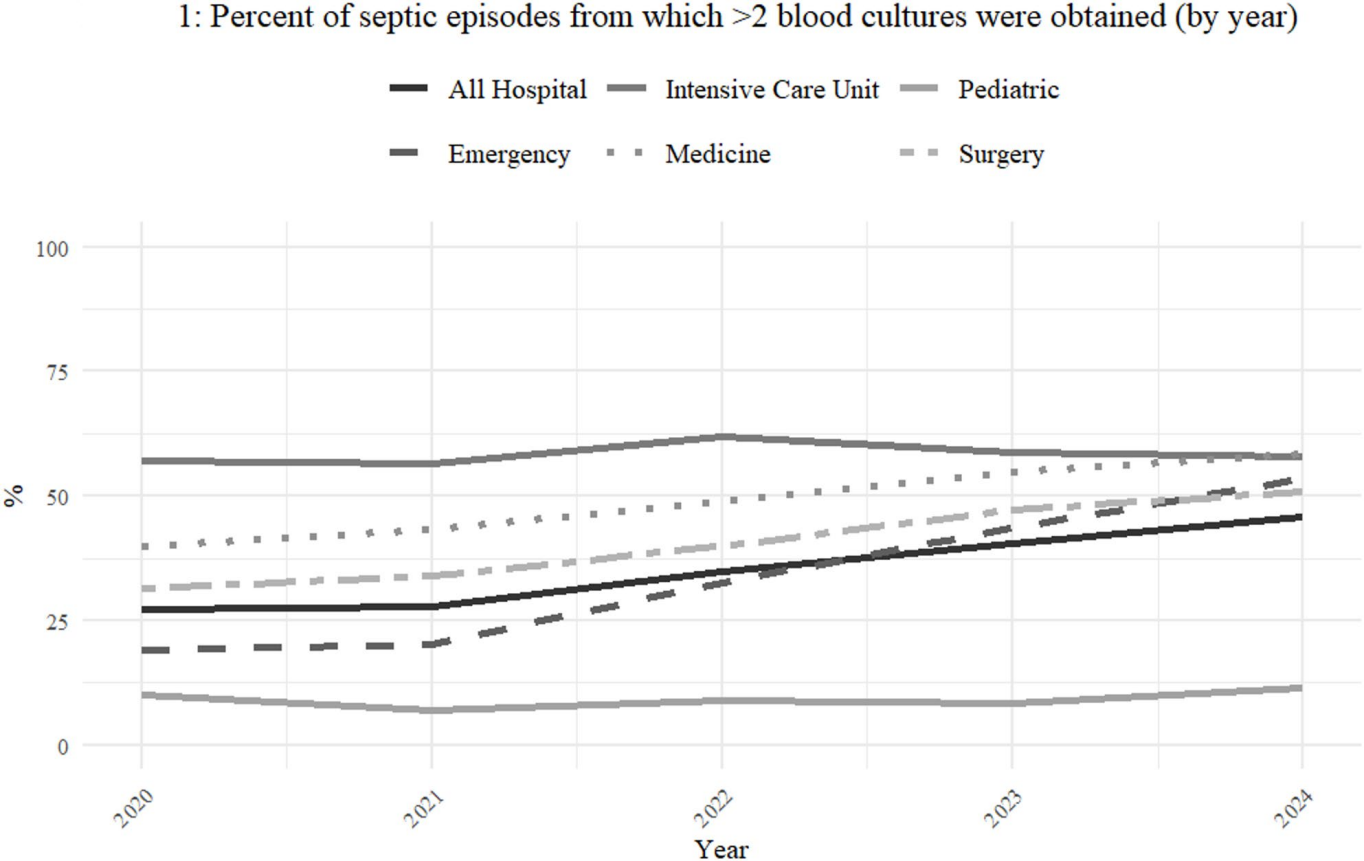
Percent of blood-culture-taking episodes from which ≥ 2 blood cultures sets were obtained (by year)

During the 5-year study (2020–2024), the percent of blood-culture-taking episodes from which two culture sets were obtained increased annually by 16% from a baseline of 27.1% in 2020, to 45.9% in 2024 (Incidence rate ratios, IRR 1.16 [95%CI 1.13–1.18], *p* < 0.001). This improvement was observed in almost all major departments, and was especially profound in the emergency department, starting at a baseline of 19.2% and increasing to 53.5% (IRR 1.33 [95%CI 1.27–1.39], *p* < 0.001)(Fig. 1; Table 2). The intensive care unit had the highest rate of obtaining two culture sets during blood-culture-taking episodes (57.2% in 2020); however, there was no significant increase over the years, as indicated by an IRR of 1. The rate of obtaining two culture sets in pediatrics was low and remained so, as a matter of policy among paediatricians in all Israeli hospitals.

**Table 2:** Annual proportion of blood-culture-taking episodes from which ≥ 2 blood cultures were obtained, by hospital wing (2020–2024), with incidence rate ratios (IRR), 95% confidence Intervals, and p-values

Wing/year	2020	2021	2022	2023	2024	IRR (95% CI)	*P* value
ED % (n/N)	19.2 (1979/10326)	20.2 (2358/11676)	32.7 (4182/12793)	43.6 (5178/11873)	53.5 (5304/9915)	1.33 (1.27–1.39)	< 0.001
ICU % (n/N)	57.2 (1376/2407)	56.6 (1416/2503)	61.9 (1353/2185)	58.7 (1026/1747)	57.8 (967/1673)	1.01 (1–1.02.02)	0.19
Medicine % (n/N)	39.8 (3795/9547)	43.3 (5442/12562)	48.9 (6847/13990)	54.9 (7819/14255)	58.5 (8387/14329)	1.11 (1.1–1.11)	< 0.001
Pediatrics % (n/N)	10.1 (730/7254)	6.9 (669/9721)	9 (885/9804)	8.3 (789/9494)	11.5 (1027/8936)	1.06 (0.94–1.19)	0.34
Surgery % (n/N)	31.4 (1083/3453)	34 (1498/4402)	40 (1956/4891)	47.2 (2075/4395)	50.8 (2386/4699)	1.14 (1.12–1.15)	< 0.001
All Hospital % (n/N)*	27.1 (9010/33306)	27.9 (11528/41282)	34.8 (15337/44052)	40.6 (17199/42325)	45.9 (18462/40191)	1.16 (1.13–1.18)	< 0.001

ED, Emergency Department; ICU, Intensive Care Unit; Medicine, Medical Departments; Surgery, General Surgical Department

*Figures do not add up to 100%, as additional cultures were obtained in out-patient settings

**Fig. 2:**
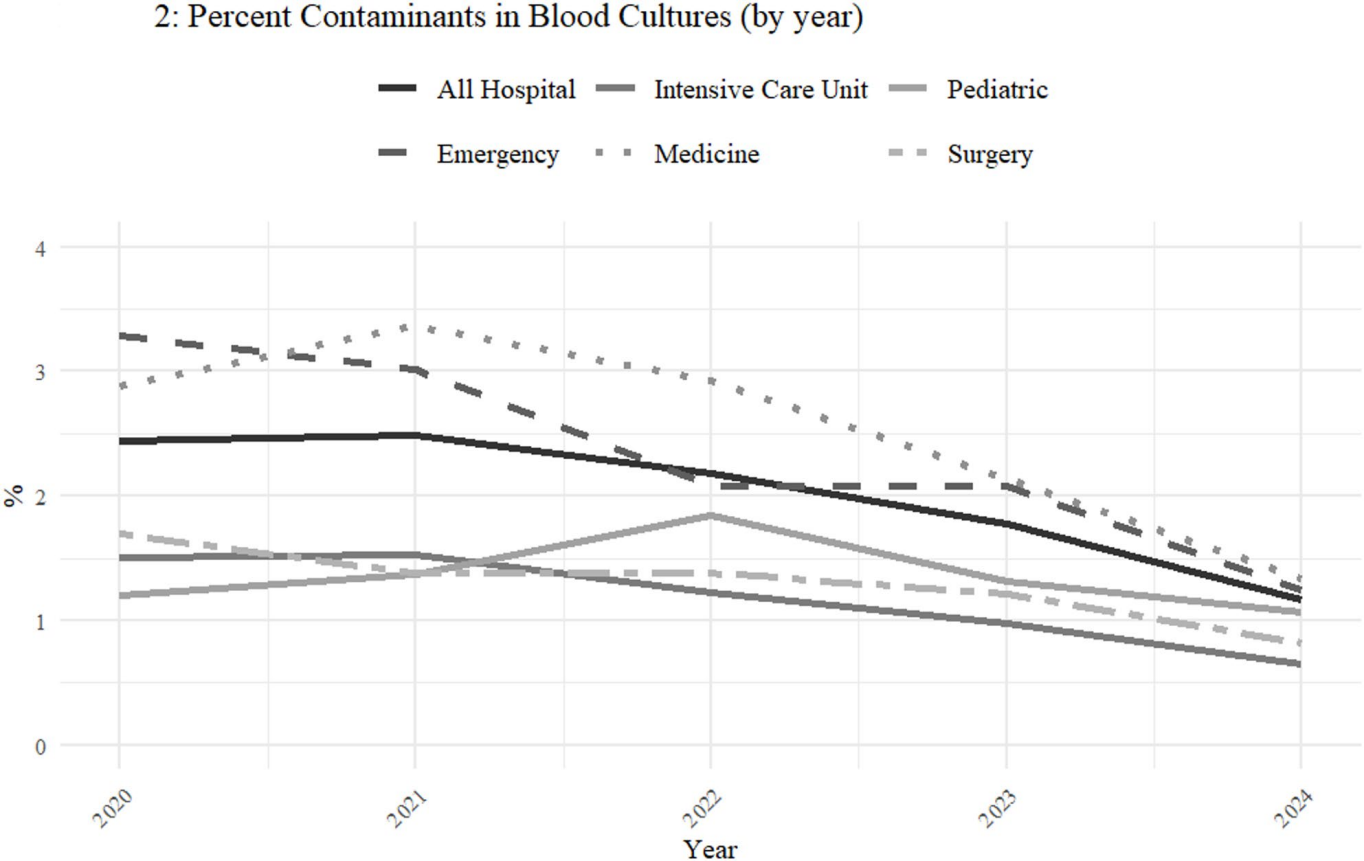
Percent of contaminated blood cultures (by year)

During the same period, the annual proportion of false-positive cultures, from which only contaminants were isolated, decreased annually by 18% from 2.4% in 2020 to 1.3% in 2024 (IRR 0.82 [95%CI 0.77–0.88], *p* < 0.001). This improvement was observed in almost all major departments, and was especially profound in the emergency department, starting at a baseline of 3.3% and decreasing to 1.56% (IRR 0.79 [95%CI 0.75–0.83], *p* < 0.001)(Fig. 2; Table 3). All departments, except pediatrics showed a clinically and statistically significant improvement over time.

**Table 3:** Annual proportion of contaminants by hospital wing (2020–2024), with incidence rate ratios (IRR), 95% confidence Intervals, and p-values

Wing/year	2020	2021	2022	2023	2024	IRR (95%CI)	*P* value
ED % (n/N)	3.29 (676/20529)	3.02 (703/23266)	2.08 (531/25479)	2.08 (492/23655)	1.56 (272/17459)	0.79 (0.75–0.83)	< 0.001
ICU % (n/N)	1.51 (72/4768)	1.53 (76/4977)	1.22 ( 53/4347)	0.98 (34/3479)	1.14 (33/2892)	0.81 (0.76–0.86)	< 0.001
Medicine % (n/N)	2.88 (545/18929)	3.36 (839/24988)	2.92 (813/27839)	2.14 (607/28392)	1.14 (357/24614)	0.8 (0.72–0.88)	< 0.001
Pediatrics % (n/N)	1.2 (163/13544)	1.37 (248/18084)	1.83 (332/18094)	1.31 (236/18041)	1.15 (157/14742)	0.93 (0.86–1.01)	0.1
Surgery % (n/N)	1.7 (116/6841)	1.38 (121/8754)	1.38 (134/9712)	1.11 (106/8728)	0.75 (61/8179)	0.84 (0.81–0.87)	< 0.001
All Hospital % (n/N)*	2.44 (1592/65230)	2.49 (2011/80688)	2.18 (1873/86021)	1.78 (1485/83393)	1.3 (895/68991)	0.82 (0.77–0.88)	< 0.001

ED, Emergency Department; ICU, Intensive Care Unit; Medicine, Medical Departments; Surgery, General Surgical Department

*Figures do not add up to 100%, as additional cultures were obtained in out-patient settings

## Discussion

Sepsis remains one of the most significant causes of infection-related mortality [11–13]. Continuing improvements in laboratory systems contribute to more rapid detection of micro-organisms and susceptibility testing, thus promoting earlier appropriate antibiotic treatment and improved outcome [12, 14–17]. Nonetheless, the human factor - optimal collection of adequate culture specimens - continues to be the major limiting factor: first, there is late and under-diagnosis due to drawing one blood culture rather than the recommended two (or more). Second, inadequate skin preparation leads to contaminated specimens [18]. The obtainment of two sets provides the double advantage of increasing blood volume (and associated chance of isolating a pathogen), as well as increasing appropriate interpretation of isolated contaminants.

A retrospective review of two decades of blood culture results in our medical center showed that two sets of blood cultures were obtained in only 32% of blood-culture-taking episodes: 27% in community-acquired episodes, and 44% in hospital-acquired episodes. Unfortunately, available diagnostic systems at the time did not allow for automated digital registration of blood culture bottle volumes, although intermittent manual weighing of bottles indicates that in up to one third of patients < 5 ml was obtained/bottle [19] rather than the required 7–10 ml. One can only speculate how many cases of bacteremia were not detected, with quite likely an inferior outcome. In addition, an overall 3.7% contamination rate during these two earlier decades indicates inadequate skin preparation, exceeding the standard target at the time of no more than 3% [20]. This high contamination rate has obviously been associated with a significant waste of laboratory resources and overuse of antibiotics in many of these patients. It is difficult to determine to which extent these data represent the state of other hospitals. However, contamination of blood cultures is a well-known problem that compromises optimal antimicrobial management and leads to a serious waste of laboratory resources [7, 8].

We employed a quasi-experimental study design [21, 22] with the two-fold object of reducing the percentage of blood culture specimens contaminated with skin organisms and increasing the percentage of blood-culture-taking episodes from which two sets of cultures were obtained at different time intervals. As shown, we were able to advance both our objects, with a sustained annual increase from 19% to 53% in procuring two sets in the emergency department (which is responsible for drawing > 30% of all blood cultures in our and most hospitals) and reducing its contamination rate from 3.3% to 1.2%. Similar improvements were observed in most other departments. More important, the annual 16% improvement in obtaining two sets of cultures was maintained throughout the study period, as was the continuous decrease in contamination rates.

There is debate in the literature regarding the need for obtaining a second blood culture set after a non-specified time interval, in case bacteremia fluctuates, or whether obtainment of the two sets at the initial venipuncture is equally effective, simultaneously saving time and inconvenience. Our data indicate that 47% of isolated organisms derive from the first set and 53% from the second set, which is considerably higher than the figure often reported in the literature (± 15%). However, these data should be viewed with caution, as in absence of recorded volumes, it is not unlikely that the triage nurse obtained smaller volumes for the first set than was done for the second sets, which are obtained by trained phlebotomists. Interestingly, the percentage of positive cultures is higher for sets drawn ≥ 72 h in the hospital than for patients seen in the emergency department (ED). This suggests more discriminate obtainment of blood cultures in the departments than in the ED, underscoring the previous observation that triage nurses may have a low threshold for obtaining blood cultures.

Interrupted time series and the quasi-experimental study design, although commonly employed in infection control studies, have the inherent disadvantage that once the intervention is discontinued the gained improvements often quickly revert to baseline levels [21–23]. Our study is, therefore, more remarkable because - except for the 1–2 short lectures with guidelines on appropriate collection of blood cultures given in all departments - the main interventions consisted of calculating the two outcome markers and sending these on a quarterly basis to all department physicians and nursing directors. The infection control practitioners (ICPs) added appropriate procurement of blood cultures to the range of issues they follow and educate about during their rounds in the departments, although according to the ICPs this issue was discussed only occasionally. We also emphasize the important contribution made by the hospital’s chief executive officer (CEO), by sending critical but constructive remarks to relevant department directors. Although at some point in time the achieved improvements are expected to level off (at > 85% of procuring two sets, and a contamination rate < 1%), the achievements have a high likelihood of sustainability – as long as results are fed back and published on a regular basis. This has become part of our regular infection control program.

Like most studies, our study has several limitations. First, to retrieve data on obtainment of two blood cultures per blood-culture-taking episode, we needed to define a blood-culture-taking episode as whenever a first blood culture was obtained and allocated up to 24 h for the obtainment of the second culture. Although we issued guidelines with indications for obtaining blood cultures, in this study we did not evaluate the reasons the included cultures were drawn. Accordingly, the triage nurse could have decided to draw a first blood culture, while the physician who subsequently examined the patient decided that the risk of sepsis was low and obviated the need for a second culture. It is quite likely that the repeat feedback led to a decrease in obtainment of a first blood culture by the triage nurse if the risk of bacteremia was considered negligible. Second, we needed to define the time window for obtaining a second culture as two consecutive calendar dates, as until recently only the day rather than the hour of procurement was registered. The two blood cultures should be obtained prior to initiation of antibiotic treatment [24–28]. It is impossible with our data set to determine the percentage of the second blood cultures which were obtained before antibiotics were started. A third limitation is the fact that we had to forgo determination of blood culture volumes as quality outcome marker, because this measure is not yet automated in our laboratory. However, as shown, the number of obtained culture sets considerably and consistently increased, suggesting an increase in overall obtained blood volume. Fourth, this was a single center study. Various medical centers likely cope with different components of inadequate blood culture procurement. It needs to be shown that the combination of the CEO’s active involvement and regular open publication of various departments’ outcome markers leads to continuing improvement in other medical centers as well. Nonetheless, several studies have shown that moderate interventions are associated with vastly improved blood culture procurement [29–31] in various settings [32]. We continue to monitor the quality of blood culture procurement by the measures outlined in this study.

In conclusion, in this study spanning five years, we demonstrated that a combination of several minor interventions led to a very significant and sustainable improvement in the quality of blood culture procurement.

## Data Availability

The data sets generated during and/or analyzed during the current study were prepared with our medical center’s electronic health record tools; the files are available from the corresponding author on request.
